# Can Post-Mortem Cartilage Reliably Preserve Ethanol? A Time-Course Comparison with Blood and Urine

**DOI:** 10.3390/toxics14080720

**Published:** 2026-08-14

**Authors:** Aleksandra Zorychta, Małgorzata Głaz, Rafał Skowronek, Joanna Nowicka, Marcin Tomsia, Elżbieta Chełmecka

**Affiliations:** 1 Department of Medical Statistic, Faculty of Pharmaceutical Sciences in Sosnowiec, Medical University of Silesia, 41-205 Sosnowiec, Poland; aleksandra.zorychta@sum.edu.pl; 2 Department of Forensic Medicine and Toxicology, Faculty of Medical Sciences in Katowice, Medical University of Silesia, 40-752 Katowice, Poland; mglaz@sum.edu.pl (M.G.); rskowronek@sum.edu.pl (R.S.); jnowicka@sum.edu.pl (J.N.)

**Keywords:** post-mortem toxicology, ethanol stability, alternative matrices, costal cartilage, intervertebral disc cartilage, room-temperature storage

## Abstract

The time interval between the collection of biological material and the performance of toxicological analyses is particularly important when the appropriate storage temperature cannot be maintained. This issue is especially relevant in the determination of ethanol concentration in specimens obtained from drivers suspected of intoxication or being under the influence of ethanol, as well as in samples collected during post-mortem examinations. The present study evaluated the effect of room-temperature storage on ethanol concentrations in post-mortem blood, urine, costal cartilage, and intervertebral disc cartilage collected from 57 cadavers. According to Polish law, blood ethanol concentrations > 0.2 mg/mL and >0.5 mg/mL were classified as “after alcohol use” and “intoxication”, respectively. Two subgroups were therefore distinguished from the study group: 27 ethanol-positive > 0.5 mg/mL (intoxicated) and 30 ethanol-negative cases—less than or equal to 0.5 mg/mL (sober). Ethanol concentrations were determined using gas chromatography with flame ionization detection (GC-FID). Statistically significant differences in blood ethanol concentration were observed in ethanol-positive individuals depending on storage time after sample collection (*p* < 0.001). Under the storage conditions used in this study, no increase in ethanol concentration was observed in samples collected from ethanol-negative individuals. In ethanol-positive individuals, ethanol concentrations decreased progressively in all analyzed matrices during room-temperature storage. Urine showed the greatest stability throughout the observation period. In both cartilage matrices, ethanol was detectable up to day 3, whereas all measurements were negative from day 4 onward. Blood ethanol concentrations also decreased during storage. By day 4, the mean concentration was below the legal intoxication threshold.

## 1. Introduction

Ethyl alcohol (ethanol) is the most widely used and legal psychoactive substance in the world. As a depressant, ethanol inhibits central nervous system (CNS) activity, primarily through interactions with GABA-A receptors and the inhibition of glutamate receptors [1,2]. This toxic xenobiotic, characterized by a low molecular weight and high water solubility, diffuses into all tissues and body fluids after absorption and metabolism [2,3]. Depending on their water content, these tissues exhibit varying alcohol concentrations compared to blood—higher in the vitreous humour or lower in costal cartilage [4,5,6]. These physicochemical properties of ethanol are of key practical importance, as they enable the use of alternative tissue samples when blood is unavailable for toxicological testing. Determining the concentration of ethyl alcohol in the body, with particular emphasis on peripheral blood analysis, is a fundamental diagnostic procedure. This is because the measured blood ethanol level determines the criminal classification of an act, allowing for an assessment of the degree of impairment of bodily functions—including psychomotor performance and behaviour—that may have directly caused or contributed to death [7,8]. Fatal intoxications with ethyl alcohol constitute the majority of cases encountered in the analysis of psychoactive substances [8,9,10,11,12,13,14,15]. For accurate post-mortem diagnosis, both the pre-analytical and analytical stages are of critical importance [16,17,18]. One of the key factors is the proper collection and storage of biological samples. The loss of a volatile analyte from the examined matrix may occur as a result of its metabolism under ante-mortem conditions or through post-mortem evaporation [19,20,21,22,23,24]. Strict adherence to established procedures protects not only the analyst from potential infection associated with biological material but also the integrity of the matrix itself [25]. Post-mortem material is often unique and cannot be re-sampled. For many years, blood and urine have remained the only widely accepted matrices in forensic toxicology [26]. Cartilaginous tissues, owing to their avascular nature and dense extracellular matrix, are less susceptible to post-mortem degradation and microbial colonization than many soft tissues. Consequently, they have attracted considerable interest in forensic genetics [27,28] and forensic epigenetics [29,30]. In contrast, their application in forensic toxicology remains limited, largely due to challenges in the use and interpretation of alternative biological matrices [2,31,32]. In the present study, we analyze the effects of the improper storage of blood, urine, and cartilaginous tissue samples at room temperature.

## 2. Materials and Methods

Biological material was collected during medico-legal autopsies performed in medical university. The study protocol was approved by the Bioethical Commission of the Medical University of Silesia in Katowice (No. KNW/0022/KB/206/18). Permission for sample collection and scientific analysis was additionally obtained from the prosecutor’s office. Femoral blood, urine, costal cartilage, and intervertebral disc specimens were obtained. All toxicological analyses were performed in an accredited forensic laboratory that successfully participates in annual external quality assurance programs. The laboratory holds a current ETB 2/26 certificate in forensic toxicology (blood alcohol concentration), valid until May 2027. (Proficiency testing is organized by ARVECON GmbH on behalf of and in cooperation with GTFCh (Gesellschaft für Toxikologische und Forensische Chemie)).

The study group comprised 27 cadavers in which ethanol was detected in blood in concentrations greater than 0.5 mg/mL. The control group consisted of 30 cadavers with concentrations in blood less than or equal to 0.5 mg/mL. The intoxication group was defined as having a blood ethanol concentration of >0.5 mg/mL, whereas concentrations below or equal to this threshold were classified as being in the sober group.

Two blood alcohol concentration thresholds were applied for data analysis. According to Polish legal regulations, a blood alcohol concentration above 0.2 mg/mL is classified as a condition after alcohol consumption. A concentration exceeding 0.5 mg/mL is considered intoxication. These thresholds have distinct legal consequences. The former is a misdemeanour, whereas the latter is a criminal offence under Polish law. The procedures for sample collection, preservation, cleaning, preparation, and toxicological analysis have been described in detail in our previous publications [31,32].

### 2.1. Sample Preparation

The inclusion criteria were as follows: (i) a statement from the forensic pathologist confirming that sample collection would not interfere with the determination of the cause of death; (ii) no objection from the prosecutor to the collection of samples and their use for scientific research; (iii) a post-mortem interval (PMI) not exceeding 9 days; and (iv) no information indicating the presence of Auto-Brewery Syndrome (ABS).

Samples were collected without the use of preservatives. Five millilitres of peripheral blood, 5 mL of urine, a costal cartilage fragment measuring approximately 5 × 3 cm, and one intervertebral disc from the L4/L5 lumbar segment were collected from each case. Only specimens with preserved gross morphology and no visible pathological or decomposition-related changes were included in this study.

Costal cartilage specimens were obtained from the lower costal arch, whereas intervertebral disc samples were collected from the lumbar L4/L5 segment. Immediately after sampling, the cartilaginous tissues were separated from the surrounding soft tissues using sterile scalpels. To minimize the risk of cross-contamination, the scalpels were replaced three times during tissue preparation. The costal cartilage and the annulus fibrosus of the intervertebral disc were then cut into smaller fragments using sterile scalpels. Fragmentation facilitated sample collection at subsequent time points, as cartilage is gradually dehydrated and hardens during storage.

**Figure 1:**
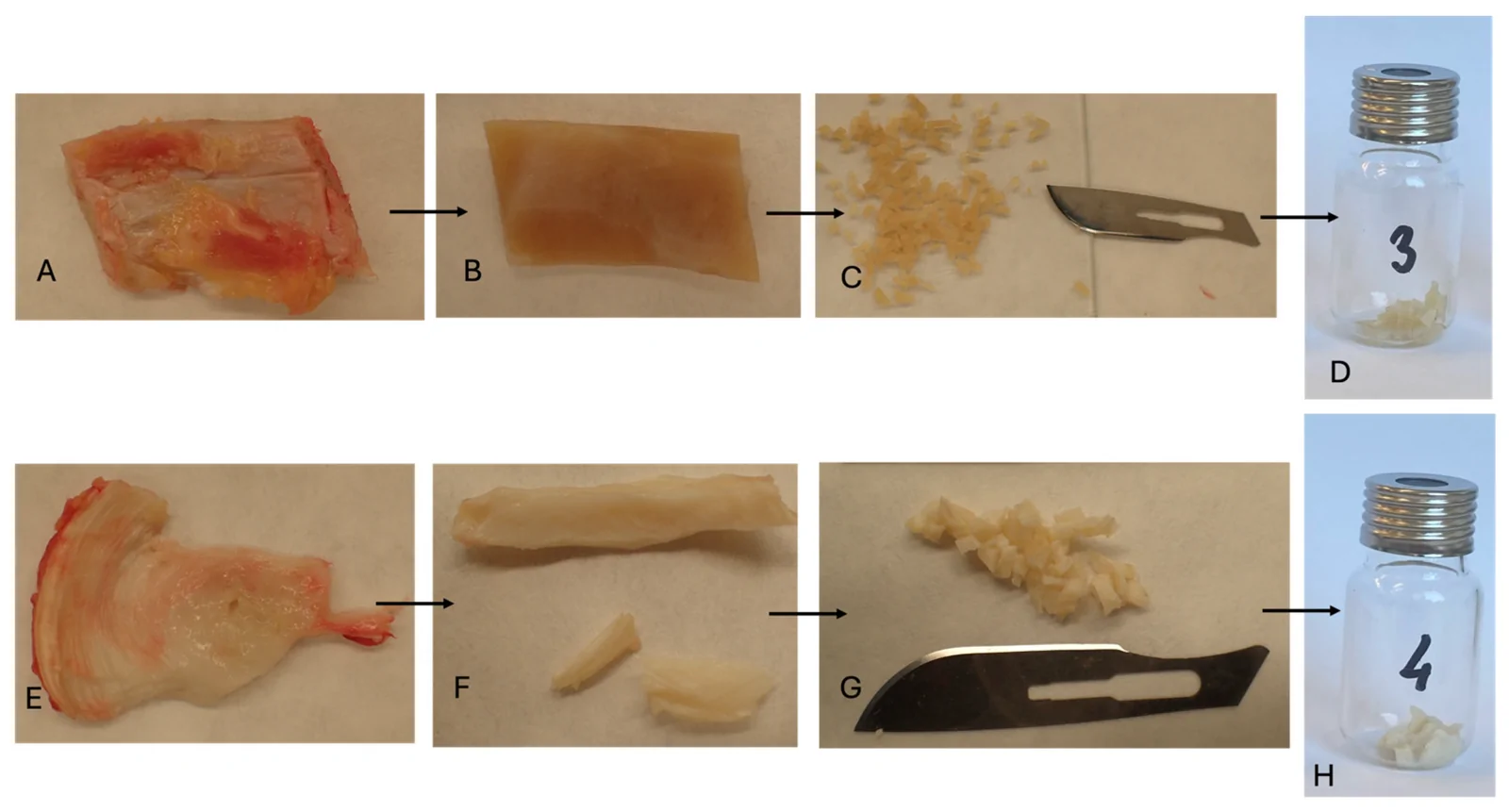
Preparation of costal cartilage (CC) and annulus fibrosus of intervertebral disc (AFID): (**A**) Cartilage as received from autopsy room. (**E**) Annulus fibrosus of intervertebral disc as received from mortuary. (**B**) Cartilage cleaned of muscle tissue. (**F**) Disc cleaned of muscle tissue. (**C**) Clean cartilage fragments to be analyzed. (**G**) Clean disc fragments to be analyzed. (**D**) Vial prepared for chromatographic analysis (**D**) with cartilage and (**H**) disc.

The processed tissue samples, together with blood and urine specimens, were transferred into separate 15 mL Falcon tubes. Individual aliquots were prepared for each planned sampling time point to avoid the repeated opening of the same container and to minimize evaporation. The tubes were stored with access to atmospheric air under conditions simulating refrigeration failure. After filling, the tubes were closed with caps, leaving a residual headspace containing atmospheric air above the sample. Storage took place in a room with monitored temperature and relative humidity using a TFA 30.5002 thermo-hygrometer (TFA Dostmann GmbH & Co. KG, Wertheim am Main, Germany). Because the material was collected over a 12-month period, the storage temperature ranged from 19.9 to 26.0 °C, and the relative humidity ranged from 30% to 60%. Femoral blood and urine samples (0.2 mL each) were collected into glass vials in accordance with established forensic toxicology guidelines [33]. For each analysis, 0.2 g of costal cartilage and 0.2 g of fibrous ring tissue were weighed and subjected to further toxicological examination. A detailed workflow of costal cartilage and intervertebral disc sample preparation is presented in Figure 1.

All specimens were analyzed at six predefined time points. The initial measurement (t0) was performed immediately after sample collection during the medico-legal autopsy. Subsequent analyses were carried out after 24 h (t1), 48 h (t2), 72 h (t3), and 96 h (t4) of storage. The final measurement (t5) was conducted 7 days after t0. Owing to weekend and public holiday constraints, the last analytical series was performed on the following Monday, corresponding to one week after the initial examination.

### 2.2. Ethanol Level Analysis with Gas Chromatography with Flame Ionization Detector (GC-FID)

Ethanol concentrations were determined using a headspace gas chromatography method based on the procedure described by Grellner and Iffland [34], with modifications introduced for the analysis of cartilaginous tissues.

Before analysis, 0.2 mL of blood or urine, or 0.2 g of costal cartilage and fibrous ring tissue from the intervertebral disc, was transferred into a headspace vial and combined with 0.2 mL of the internal standard solution.

Chromatographic measurements were performed using a Focus GC gas chromatograph equipped with a Triplus autosampler, FID (Thermo Fisher Scientific, Inc., Waltham, MA, USA), and Rtx^®^–BAC2 column (30 m × 0.53 mm i.d., 2.0 μm film thickness; Restek, Bellefonte, PA, USA). The oven temperature was initially maintained at 45 °C for 5 min, then increased to 80 °C at a rate of 10 °C/min and held for 1 min. The injector and detector temperatures were set at 200 °C and 250 °C, respectively. Helium was used as the carrier gas at a constant flow rate of 5.0 mL/min. Tert-butyl alcohol served as the internal standard.

**Figure 2:**
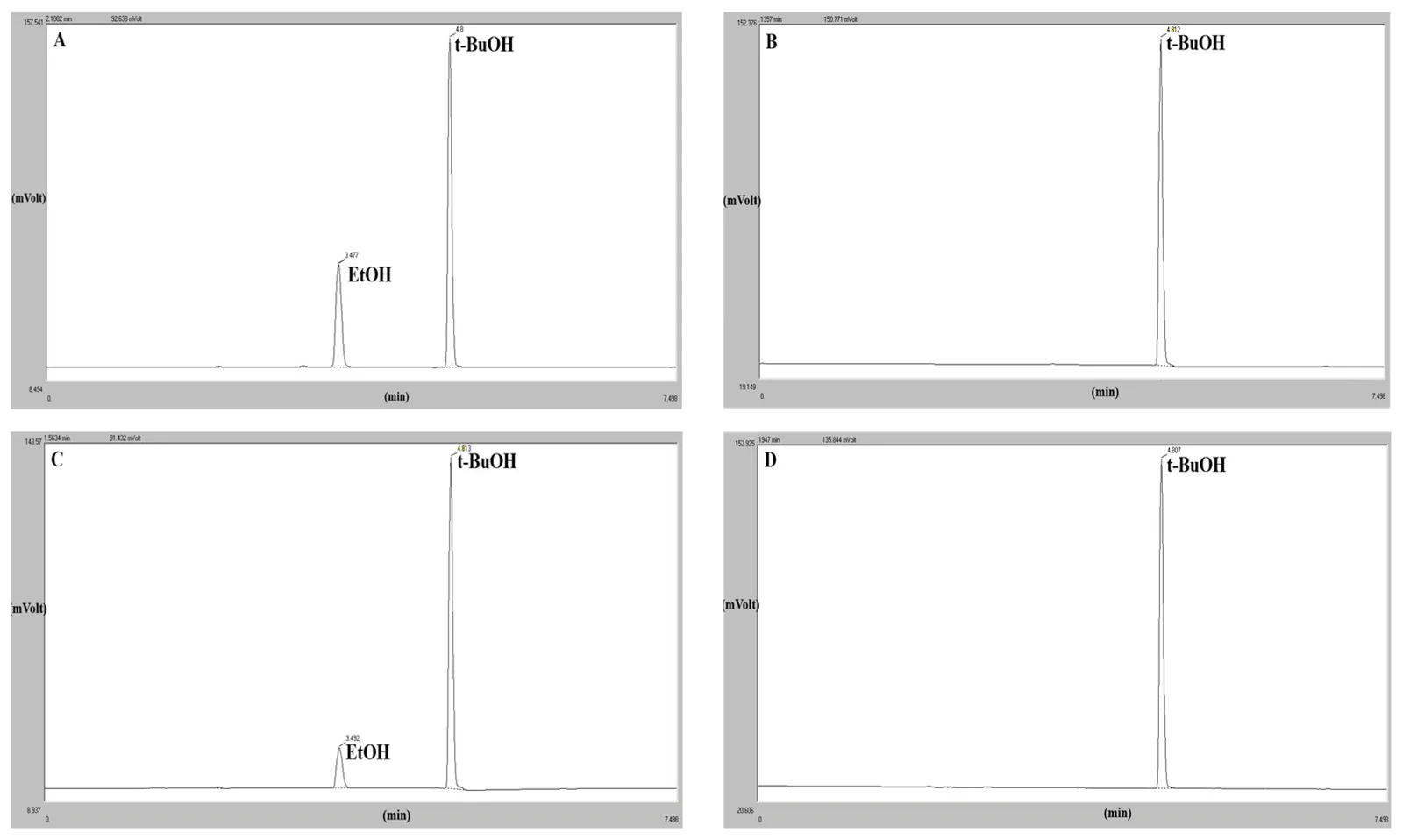
Example chromatograms (**A**) with 0.82 mg/mL ethanol content and (**B**) with 0.0 mg/mL ethanol of costal cartilage (CC) and (**C**) with 0.22 mg/mL ethanol content and (**D**) with 0.0 mg/mL ethanol of the annulus fibrosus of the intervertebral disc (AFID). Abbreviations: EtOH—ethanol; t-BuOH—tert-butanol, which is internal standard (IS).

Quantification was based on an eight-point calibration curve covering concentrations of 0, 0.1, 0.2, 0.5, 0.8, 1.0, 2.0, and 3.0 mg/mL or mg/g. Linearity was confirmed throughout the entire calibration range. The limit of detection was 0.05 mg/mL (or mg/g), whereas the limit of quantification was established at 0.1 mg/mL (or mg/g) for all analyzed matrices. Figure 2 presents example chromatograms of negative and positive samples of costal cartilage (CC) and annulus fibrosus of the intervertebral disc (AFID).

### 2.3. Data Processing

Quantitative determinations of ethanol in blood, urine, costal cartilage, and intervertebral disc samples were performed using headspace gas chromatography to detect the presence of alcohol. The chromatographic data obtained during the analysis were processed using ChromCard software, version 2.3.3. Calibration curves were constructed based on the relationship between the ratio of the ethanol peak area to the internal standard (IS) peak area and the analyte concentration, using linear regression. The resulting calibration equations were used to determine the concentrations of ethyl alcohol in the analyzed samples. The results of ethanol concentration determinations in the examined autopsy specimens, obtained at specified time points, were compiled together with data from the case files in Microsoft Excel and then subjected to statistical analysis.

### 2.4. Statistical Analysis

The Shapiro–Wilk test was used to assess the normality of the distribution. Normal distributions were characterized by means and standard deviation (m ± s), while non-normal distributions were described by medians and lower and upper quartiles: Me(Q_1_–Q_3_). Additionally, the minimum and maximum values were included in the tables to illustrate the range of analyzed ethyl alcohol concentrations. The Spearman rank correlation coefficient or Pearson’s linear correlation coefficient was used to assess the relationship between alcohol concentrations in different matrices. To determine the differences between the sober and intoxicated groups or gender, for normal-distributed variables, the t-Student test for independent samples was used, and for skewed data, the Mann–Whitney test was used. A single-sample *t*-test or the Wilcoxon signed-rank test was used for comparisons with the limit value.

Statistical analyses of alcohol concentrations over time were performed exclusively for intoxicated individuals. Variations in alcohol levels across the timeline were evaluated using a repeated measures Analysis of Variance (RM-ANOVA). The assumption of sphericity was verified using Mauchly’s test; in instances where this assumption was violated, degrees of freedom were adjusted using the Greenhouse–Geisser correction. Statistical outputs were reported using the F statistic, effect and error density of freedom and effect size (partial eta-squared): *F(df_effect_, df_error_) = F-value*, *p = p-value*, *η^2^ = value*. To explore specific differences between individual time points, planned contrast analyses were conducted.

The statistical model incorporated six distinct time points for blood and urine matrices and four time points for cartilage and AFID matrices.

Calculations and the creation of figures were performed in Statistica (TIBCO Software Inc., Palo Alto, CA, USA (2017)). Statistica (data analysis software system), version 13) and PQStat (PQStat Software (2025). PQStat v.1.8.6.126, Poznan, Poland). The tests were two-sided. The results were considered statistically significant at *p* < 0.05.

## 3. Results

### 3.1. Descriptive Statistics

A total of 57 cadavers, ranging in age from 7 days to 79 years, were included in this study (mean ± standard deviation: 41.8 ± 16.2 years). Autopsies were performed between 1 and 9 days after death. The post-mortem interval (PMI) was 4.0 days (median; lower quartile–upper quartile: 3.0–5.0 days). Mean body height was 173.4 ± 10.8 cm, and mean body weight was 77.3 ± 20.5 kg. The descriptive characteristics of the study population, together with the ethanol concentrations measured in four biological matrices, are presented in Table 1.

The coefficients of variation (Vs) were calculated for all analyzed matrices and compared with the value obtained for blood (blood: Vs = 122%). No significant differences were found between blood and urine (urine: Vs = 123%; F = 0.990; *p* = 0.960), rib cartilage (Vs = 124%; F = 0.987; *p* = 0.959), or annulus fibrosus of the intervertebral disc (Vs = 130%; F = 0.953; *p* = 0.857).

Based on the initial blood ethanol measurements, individuals were classified as sober or intoxicated using the legal threshold of blood ethanol concentration of 0.5‰. Twenty-seven individuals (47%) were classified as intoxicated. For the purposes of this study, “sober” refers to individuals with an initial blood ethanol concentration below or equal to the intoxication threshold of 0.5‰ and does not necessarily imply the complete absence of analytically detectable ethanol. No association was found between sex and intoxication status (χ^2^ = 0.198; *p* = 0.656). No significant differences were observed between intoxicated and sober individuals with respect to age (t = 1.114; *p* = 0.270) or post-mortem interval (PMI) (t = 0.007; *p* = 0.994).

For comparisons between the sober and intoxicated groups across all analyzed matrices, the vast majority of ethanol concentrations in individuals classified as sober were equal to zero. Therefore, only median values of 0.0 were reported for these data in the table. Consequently, a one-sample *t*-test (comparison with 0.5‰) was performed only for intoxicated individuals. The test statistics derived from this analysis are indicated by an asterisk in Table 2.

Men constituted the majority of the study population (45 individuals, 79%). No sex-related differences were observed for age (t = 1.451; *p* = 0.153) or post-mortem interval (PMI) (z = 1.935; *p* = 0.053). Ethanol concentrations measured at autopsy did not differ significantly between sexes in blood (z = 0.500; *p* = 0.617), urine (z = 0.241; *p* = 0.810), rib cartilage (z = 0.241; *p* = 0.810), or intervertebral disc tissue (z = 0.059; *p* = 0.953). Detailed results are presented in Supplementary Table S1.

### 3.2. Analyses at Baseline (t0)

As the analyses presented above showed that the entire cohort could be treated as a single group, all participants (N = 57) were analyzed together. Comparisons were performed for ethanol concentrations at t0 across different matrices. Based on their physical state, the matrices were divided into two groups: liquid matrices (blood and urine) and solid matrices (CC and AFID).

No differences in ethanol concentration were observed between blood and urine measurements (z = 0.00; *p* = 1.000). Likewise, no differences were found between CC and AFID measurements (z = 0.00; *p* = 1.000) (Supplementary Figure S1).

In forensic investigations, the determination of sobriety based on blood ethanol concentration is considered the gold standard. Therefore, ethanol concentrations measured in the different matrices were compared with those determined in blood.

### 3.3. PMI

PMI values were compared with ethanol concentrations in all analyzed matrices. In the study group as a whole, no association was found between PMI and ethanol concentration in blood (r_S_ = −0.054; *p* = 0.690), urine (r_S_ = 0.000; *p* = 0.999), cartilage (r_S_ = −0.030; *p* = 0.829), or intervertebral disc tissue (r_S_ = −0.029; *p* = 0.847).

An additional analysis was performed for the subgroup classified as intoxicated (*n* = 27). No significant association was observed between PMI and ethanol concentration in blood (r = −0.251; *p* = 0.207), cartilage (r = −0.116; *p* = 0.573), or intervertebral disc tissue (r = −0.271; *p* = 0.210). In contrast, a moderate negative correlation was found between PMI and urine ethanol concentration (r = −0.542; *p* < 0.05).

### 3.4. Correlation Analysis of Ethanol Concentrations in Different Matrices Relative to Blood

**Figure 3:**
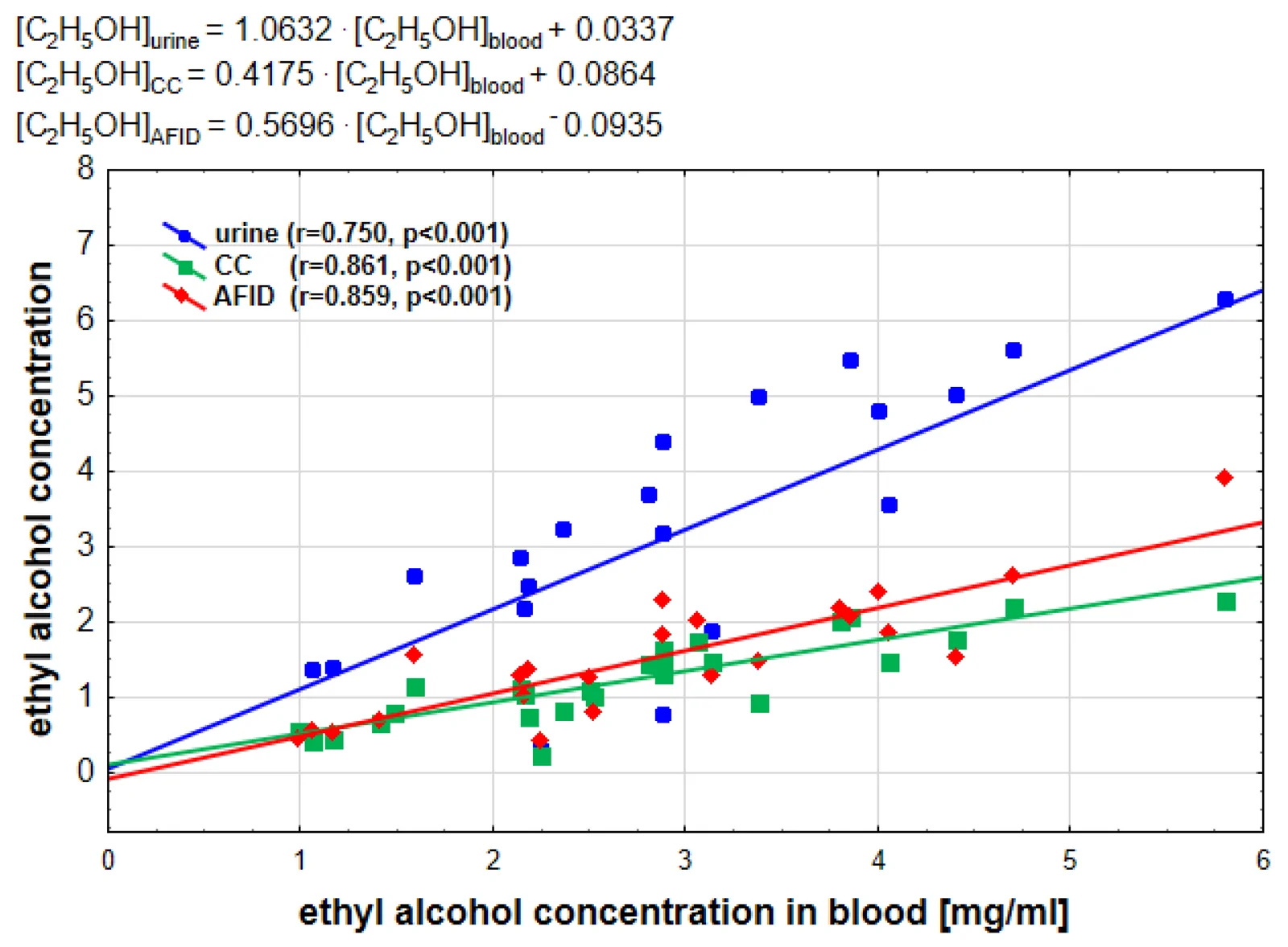
Correlation between ethanol concentration in blood and ethanol concentration in urine (blue line), CC (green line) and AFID (red line). CC—Costal Cartilage; AFID—Annulus Fibrosus of Intervertebral Disc.

Statistically significant, near-perfect positive correlations were found between blood ethanol concentration and ethanol concentration in urine (*n* = 42; r_S_ = 0.936; *p* < 0.001), cartilage (*n* = 55; r_S_ = 0.973; *p* < 0.001), and intervertebral disc tissue (*n* = 48; r_S_ = 0.966; *p* < 0.001). For further analysis, only cases classified as intoxicated according to the sobriety classification system (blood ethanol concentration > 0.5‰) were included. Within this subgroup, statistically significant strong positive correlations were observed between blood ethanol concentration and ethanol concentration in urine (*n* = 21; r = 0.750; *p* < 0.001), cartilage (*n* = 26; r = 0.861; *p* < 0.001), and intervertebral disc tissue (*n* = 23; r = 0.859; *p* < 0.001) (Supplementary Table S2, Figure 3).

### 3.5. Temporal Changes in Ethyl Alcohol Concentration According to Matrix Type and Sobriety Classification

For all analyzed matrices, cases were classified as either sober or intoxicated according to the blood ethanol results. Blood measurements were used as the reference standard, as they constitute the legal basis for the assessment of sobriety.

#### 3.5.1. Analysis of Samples Collected from Sober Individuals over Time

Among individuals classified as sober on the basis of blood ethanol analysis, ethanol concentrations were measured over time in blood, urine, cartilage, and intervertebral disc tissue for selected cases. The blood ethanol results are presented in Table 3. Observed ethanol concentrations at each sampling time were compared with the reference value of 0.0‰. In individuals classified as sober, no significant differences from 0.0‰ were found between days 0 and 3. Ethanol concentrations during this period were also significantly lower than 0.5‰. On days 4 and 7, all blood samples were negative for ethanol.

A similar analysis was performed for ethanol measurements in urine, cartilage, and intervertebral disc tissue over time. The results are presented in Table 4. For urine samples from individuals classified as sober, ethanol concentrations at all time points did not differ from 0.00. This indicates that no post-mortem increase in ethanol concentration occurred. For CC samples, ethanol concentrations were compared with the reference value of 0.0 mg/g. In individuals classified as sober, no significant differences were observed between days 0 and 2 (all *p* = 1.000). At later time points, both the minimum and maximum values recorded for costal cartilage and intervertebral disc tissue indicated the absence of ethanol in the analyzed samples.

#### 3.5.2. Analysis of Samples Collected from Intoxicated Individuals over Time

**Figure 4:**
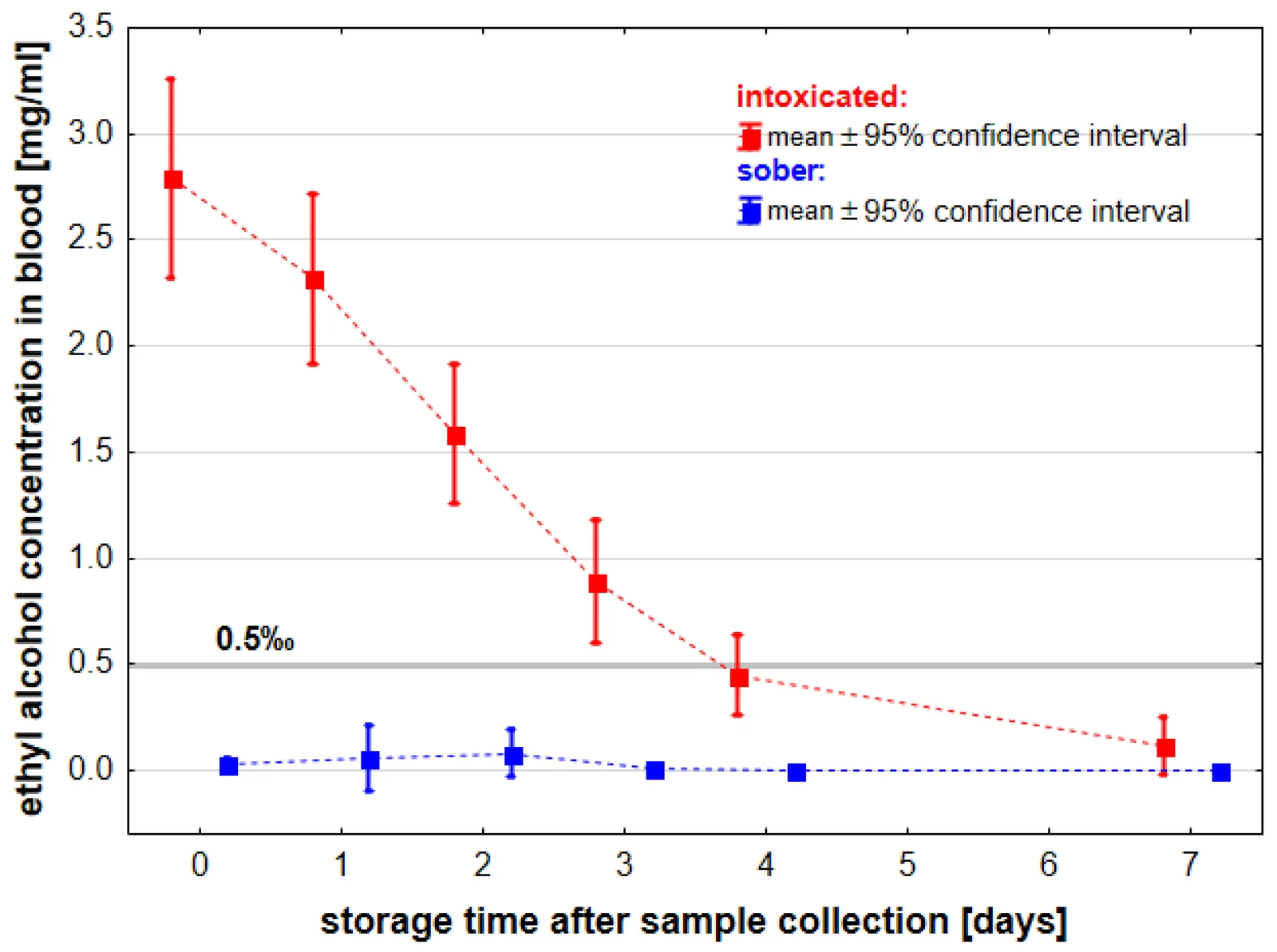
Ethyl alcohol concentration in blood [mg/mL] as a function of storage time after sample collections [days] in individuals classified as intoxicated (N = 27, red) and sober (N = 30, blue). Markers indicate mean values, with 95% confidence intervals shown. For clarity, mean values are connected with a dashed line. The grey line indicates the threshold used for determining sobriety.

Statistically significant differences in blood ethanol concentration were observed in intoxicated individuals depending on the time of sampling (F(1.48; 38.46) = 85.487, *p* < 0.001, η^2^ = 0.767). Detailed concentrations are presented in Table 5, while the results of pairwise comparisons are shown in Table 6. Ethanol concentration decreased over time. By day 7, values fell below the legal threshold used for the determination of sobriety (<0.5‰). This point is marked by a red line in the figures below. In addition, as in the sober group, the results were compared with the reference values of 0.0 and 0.5‰. On all study days except day 7, urine ethanol concentrations were higher than 0.0. From day 7 onwards, they did not differ significantly from 0.0 and were clearly below the legal alcohol limit. Figure 4 below also includes the results from both intoxicated and sober individuals for comparison.

**Figure 5:**
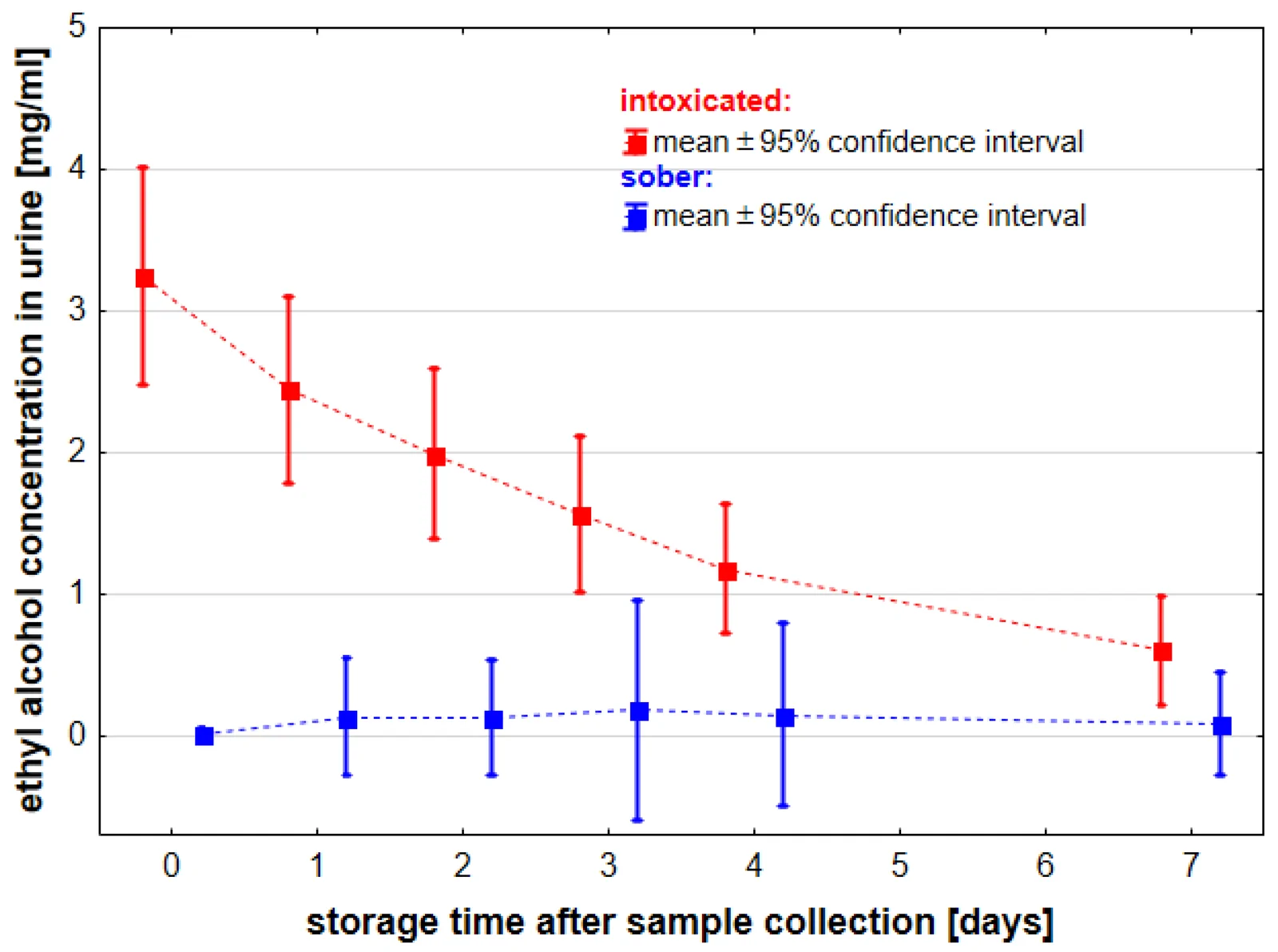
Ethyl alcohol concentration in urine [mg/dL] as a function of storage time after sample collections [days] in individuals classified as intoxicated (N = 27, red) and sober (N = 30, blue). Markers indicate mean values, with 95% confidence intervals shown. For clarity, mean values are connected with a dashed line.

As observed for blood, ethanol concentrations in urine also changed significantly over time (F(1.25; 21.18) = 44.417; *p* < 0.001, η^2^ = 0.723). Urine ethanol concentrations decreased progressively at each sampling point (Table 6). Comparison with the reference value of 0.0 showed that ethanol concentrations remained above this level throughout the study period (Table 7 and Figure 5).

**Figure 6:**
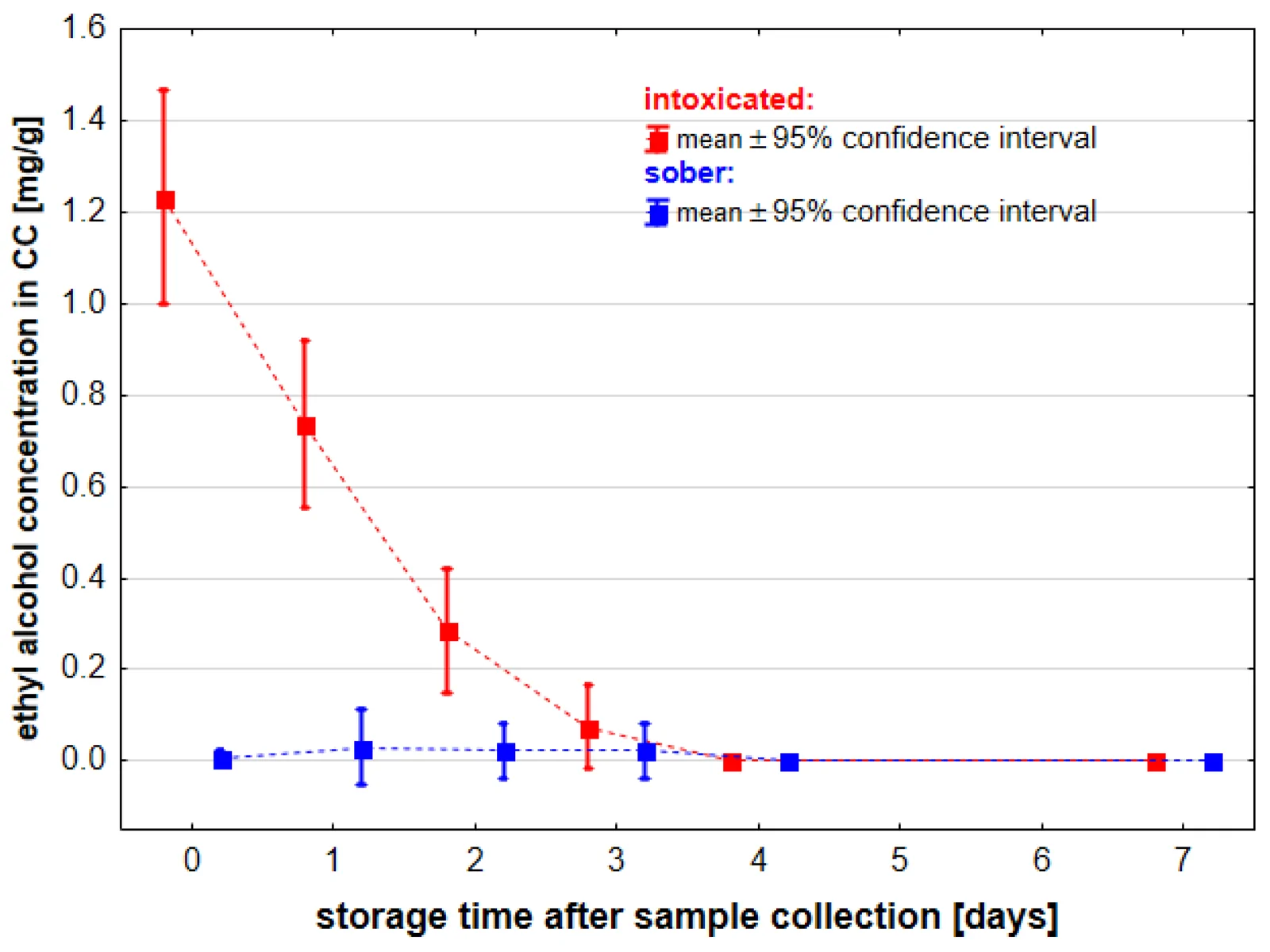
Ethyl alcohol concentration in costal cartilage [mg/g] as a function of storage time after sample collections [days] in individuals classified as intoxicated (N = 27, red) and sober (N = 30, blue). Markers indicate mean values, with 95% confidence intervals shown. For clarity, mean values are connected with a dashed line.

For costal cartilage (CC), ethyl alcohol concentrations were zero on days 4 and 7. Therefore, analyses in intoxicated individuals were restricted to days 0, 1, 2, and 3. Significant differences in ethanol concentration were found across these time points (F(1.69; 42.36) = 73.097; *p* < 0.001, η^2^ = 0.745). Pairwise comparisons showed significant differences between days 0, 1, 2, and 3. No difference was observed between days 2 and 3 (*p* = 0.073) (Table 6). From day 4 onwards, Figure 6 shows a plateau at 0.0 mg/g (Figure 6).

When compared with the reference value of 0.0 mg/g, ethanol concentrations differed significantly on day 0 (*p* < 0.001), day 1 (*p* < 0.001), and day 2 (*p* < 0.001). By day 3, this difference was no longer significant (*p* = 0.109). All subsequent measurements were 0.0 mg/g.

**Figure 7:**
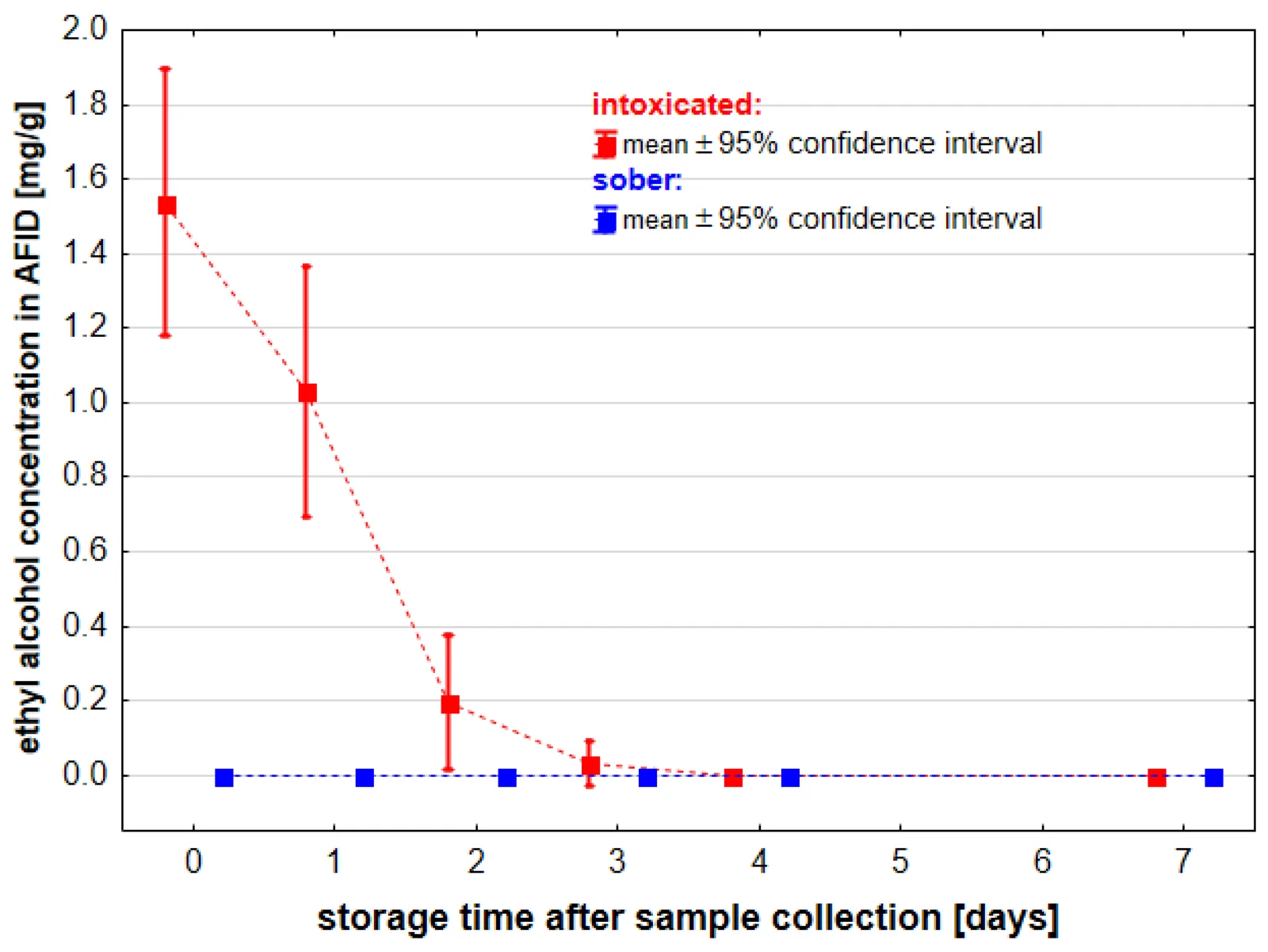
Ethyl alcohol concentration in AFID [mg/g] as a function of storage time after sample collections [days] in individuals classified as intoxicated (N = 27, red) and sober (N = 30, blue). Markers indicate mean values, with 95% confidence intervals shown. For clarity, mean values are connected with a dashed line.

A similar pattern was observed for AFID (F(1.41; 29.70 = 55.256; *p* < 0.001, η^2^ = 0.725). As ethyl alcohol concentrations were also zero on days 4 and 7, analyses were limited to days 0, 1, 2, and 3 (Table 6 and Table 7). Significant differences in ethanol concentration were found across these time points (*p* < 0.001). Pairwise comparisons showed significant differences between days 0, 1, 2, and 3, whereas no difference was detected between days 2 and 3 (*p* = 0.702) (Table 6). From day 4 onwards, a plateau at 0.0 mg/g was observed (Figure 7).

## 4. Discussion

Blood ethanol analysis remains the reference method for assessing intoxication in forensic practice and is recognized as the legal gold standard for the interpretation of alcohol-related findings. Urine is also widely accepted as a valuable supplementary matrix, particularly in post-mortem investigations. Nevertheless, alternative biological materials continue to attract attention, especially in cases involving advanced decomposition, extensive trauma (when, despite the availability of muscle tissue, uncertainty remains regarding the potential occurrence of contamination), severe blood loss, or difficulties in obtaining conventional specimens [26,35]. For this reason, the present study evaluated ethanol concentrations in costal cartilage (CC) and the annulus fibrosus of the intervertebral disc (AFID), with particular emphasis on the effect of sample storage time on analytical results.

The first objective was to determine whether ethanol concentrations measured in these tissues reflect concentrations observed in blood. The results demonstrated a very strong relationship between blood ethanol concentration and concentrations measured in both cartilage-based matrices. Nearly perfect positive correlations were observed between blood and CC ethanol concentrations, as well as between blood and AFID ethanol concentrations. These associations remained strong when the analysis was restricted to intoxicated individuals. Similar findings were reported by Tomsia et al. [32], who demonstrated close agreement between ethanol concentrations measured in costal cartilage, blood and urine, suggesting that cartilage may provide useful toxicological information when conventional matrices are unavailable [2]. Comparable observations have also been reported for other volatile compounds analyzed in costal cartilage [32].

The findings obtained for AFID are of particular interest. To date, intervertebral disc tissue has been investigated primarily in the context of forensic identification and DNA analysis in severely decomposed or charred remains [27]. Information regarding ethanol determination in this tissue remains limited. The strong correlations observed in the present study indicate that AFID may represent a valuable alternative matrix for post-mortem ethanol analysis. This observation is further supported by studies investigating fatal methanol poisoning, in which strong relationships between alcohol concentrations in blood, costal cartilage and intervertebral disc cartilage were also demonstrated [36]. These findings suggest that cartilage tissues may have broader applications in forensic toxicology than previously recognized.

An important aspect of the present study was the distinction between post-mortem interval (PMI) and sample storage time. The PMI refers to the period between death and autopsy, whereas the temporal analyses performed in the present study assessed changes occurring after sample collection. No significant associations were observed between PMI and ethanol concentrations in blood, urine, CC or AFID. Similar conclusions have been drawn in recent reviews, which indicate that the PMI alone is often a poor predictor of post-mortem ethanol behaviour because numerous biological and environmental factors influence ethanol concentrations after death [16]. Likewise, Kugelberg and Jones emphasized that the interpretation of post-mortem alcohol findings requires the consideration of multiple variables, including microbial activity, specimen type and storage conditions rather than the PMI alone [36].

However, distinct changes were observed during storage at room temperature. Ethanol concentrations decreased progressively in all analyzed matrices throughout the observation period. This pattern was the most evident among individuals classified as intoxicated.

Urine exhibited the greatest stability. Although ethanol concentrations gradually declined with time, ethanol remained detectable throughout the seven-day observation period. These findings support the long-established role of urine as a stable and informative matrix in forensic alcohol investigations. Previous studies have reported relatively limited changes in urinary ethanol concentrations during storage, particularly when specimens are appropriately preserved [37]. The results obtained in the present study are consistent with these observations and indicate that urine retains diagnostic value longer than the cartilage-based matrices examined here.

More pronounced changes were observed in costal cartilage and AFID. In both tissues, ethanol concentrations decreased up to day 3, whereas all measurements were negative from day 4 onward. These findings suggest that both matrices retain diagnostic value shortly after collection but become substantially less informative during prolonged storage. Consequently, CC and AFID may be useful for immediate post-mortem toxicological analysis, whereas delayed testing should be interpreted with caution.

The observed reduction in ethanol concentration during storage at room temperature is consistent with previous studies examining ethanol stability in biological specimens. Ferrari et al. demonstrated a progressive decrease in blood ethanol concentration during storage and showed that the magnitude of change depended on storage conditions and microbial contamination [22]. Similar observations were reported by Shan et al. [23]. Laurens et al. identified storage temperature as one of the most important pre-analytical factors affecting ethanol stability and highlighted the importance of appropriate sample preservation [38].

Several studies have demonstrated that ethanol concentrations may decline substantially when samples are stored at room temperature, whereas refrigerated specimens generally exhibit greater stability. Ferrari et al. observed progressive reductions in ethanol concentration over time under less favourable storage conditions [22]. In contrast, Olsen and Hearn [20], as well as Sutlovic et al. [19], reported relatively stable ethanol concentrations in properly preserved post-mortem blood samples stored under refrigerated conditions. Compared with these findings, the rapid decline observed in CC and AFID suggests that cartilage tissues may be more susceptible to storage-related ethanol loss than blood. This difference may be related to tissue composition, lower water content, diffusion characteristics, or increased ethanol evaporation from the analyzed tissues.

An equally important finding was the absence of increasing ethanol concentrations in samples obtained from individuals classified as sober. Across all analyzed matrices, ethanol concentrations remained at zero or did not differ significantly from zero throughout the observation period. It should be emphasized, however, that this finding applies only to the conditions investigated, namely storage at room temperature. These findings provide no evidence of post-mortem ethanol formation during storage. This observation is relevant because endogenous alcohol production remains one of the major challenges in the forensic interpretation of post-mortem toxicological results. O’Neal and Poklis emphasized that post-mortem ethanol production is strongly influenced by microbial activity, decomposition and storage conditions [39]. The absence of an increase in ethanol concentration in the present study suggests that such processes did not significantly affect the analyzed samples during the seven-day observation period. However, their occurrence beyond this observation period cannot be excluded.

The practical implications of these findings are twofold. First, both costal cartilage and AFID showed strong positive associations with blood ethanol concentrations at the time of collection and may therefore serve as useful alternative matrices when conventional specimens are unavailable. Second, both tissues demonstrate considerably lower ethanol stability during storage than urine and, according to available evidence, probably lower stability than properly preserved blood samples. Consequently, their forensic value appears the greatest when analyses are performed shortly after collection.

Several limitations should be acknowledged. The number of intoxicated individuals was relatively small, and only one set of storage conditions was evaluated. Another limitation was the lack of comparison with other alternative biological matrices, such as vitreous humour and muscle tissue, which are already used in forensic practice when blood is unavailable or cannot be adequately collected. Additional studies involving larger populations and controlled comparisons of different temperatures and preservation protocols, as well as other alternative matrices, would improve the understanding of ethanol stability in cartilage tissues. Such investigations may help define the optimal conditions for the forensic use of these matrices. An equally important factor in obtaining reliable qualitative and quantitative results for the determination of ethanol concentration in body fluids and other tissues is the proper selection of the analytical method. Preference is given to methods that are simple, widely used, and validated in interlaboratory proficiency testing [40], which increases the confidence in obtaining accurate, precise, and reproducible results [41]. One such method, routinely used worldwide, is headspace gas chromatography (HS-GC). In accordance with current recommendations and generally accepted practice, the optimal sample for post-mortem ethanol concentration determination is blood drawn from the femoral vein, which is highly resistant to changes occurring during post-mortem processes [36]. However, this material is not always available; therefore, it is necessary to conduct research on alternative biological materials that allow for the assessment of ethanol concentration at the time of death. It is equally important to conduct studies evaluating the stability of this xenobiotic in alternative biological materials, which was the focus of the present study.

In conclusion, the present study demonstrated very strong relationships between ethanol concentrations measured in blood and those measured in costal cartilage and AFID. Both tissues may serve as useful alternative matrices for post-mortem ethanol determination. However, ethanol concentrations in these tissues decline rapidly during storage and become undetectable after several days. In contrast, urine showed substantially greater stability over time. These findings indicate that CC and AFID are the most suitable for analyses performed shortly after sample collection and should be interpreted cautiously when prolonged storage cannot be avoided.

## 5. Conclusions

The present study demonstrated strong positive relationships between ethanol concentrations measured in blood and those measured in costal cartilage (CC) and annulus fibrosus of the intervertebral disc (AFID), supporting the potential use of these tissues as complementary alternative matrices for post-mortem ethanol analysis when conventional specimens are unavailable.

Under the room-temperature storage conditions investigated in this study, ethanol concentrations decreased progressively in blood, urine, CC, and AFID. Urine showed the greatest stability over the seven-day observation period, whereas ethanol in CC and AFID became undetectable from day 4 onwards. These findings indicate that CC and AFID may be the most informative when analyzed shortly after collection and that delayed analysis should be interpreted with caution.

No clear increase in ethanol concentration was observed in samples from individuals classified as sober under the conditions investigated. However, this finding does not exclude the possibility of endogenous post-mortem ethanol formation under other circumstances. Therefore, the present results should be interpreted as providing no evidence of measurable ethanol formation under the experimental conditions used, rather than as demonstrating that endogenous ethanol production cannot occur.

Importantly, a negative ethanol result obtained from CC or AFID after prolonged room-temperature storage should not be interpreted as evidence of sobriety at the time of death. The interpretation of delayed negative results should take into account the time elapsed since sampling, storage conditions, PMI, the availability of other biological matrices, and the overall forensic context.

Further studies involving larger study populations and controlled comparisons of room-temperature, refrigerated, and frozen storage are warranted. Future research should also include additional alternative matrices, particularly muscle tissue and vitreous humour, to better define the complementary role and limitations of CC and AFID in post-mortem ethanol analysis.

## Figures and Tables

**Table 1:** The descriptive characteristics of the study population, together with the ethanol concentrations measured in four biological matrices.

Variable	N	M	s	Me	Q_1_	Q_3_	min	max	As
**Age (years)**	54	41.8	16.2	42.0	31.0	51.0	0.0	79.0	−0.05
**PMI (days)**	57	4.0	1.9	4.0	3.0	5.0	1.0	9.0	0.55
**High (cm)**	55	173.4	10.8	172.0	165.0	182.0	151.0	195.0	0.03
**Mass (kg)**	55	77.3	20.5	74.0	63.0	85.0	42.0	136.0	0.82
**Ethyl alcohol concentration in:**									
**Blood (mg/mL)**	57	1.35	1.61	0.32	0.00	2.52	0.00	5.80	0.87
**Urine (mg/mL)**	42	1.63	2.02	0.33	0.00	3.17	0.00	6.30	0.90
**CC (mg/g)**	55	0.59	0.73	0.00	0.00	1.13	0.00	2.30	0.89
**AFID (mg/g)**	48	0.74	0.96	0.00	0.00	1.43	0.00	3.90	1.21

Legend: N—number of cases; M—mean; s—standard deviation; Me—median; Q_1_—lower quartile; Q_3_—upper quartile; min—minimum value; max—maximum value; As—skewness coefficient; CC—costal cartilage; AFID—annulus fibrosus of the intervertebral disc.

**Table 2:** A comparison of the analyzed variables according to sobriety status as defined by the legal blood ethanol threshold.

Variable	Sobriety Status		t/t*	*p*
	≤0.5 mg/mL N = 30	>0.5 mg/mL N = 27		
**Age** **(** **years** **)**	39.4 ± 18.6	44.0 ± 16.1	1.114	0.270
**PMI (d** **ays** **)**	4.0 ± 1.8	4.0 ± 2.0	0.007	0.994
**Ethyl alcohol concentration in:**				
**Blood (mg/mL)**	0.00	2.79 ± 1.19	12.182 *	<0.001
**Urine (mg/mL)**	0.00	3.24 ± 1.70	8.774 *	<0.001
**CC (mg/g)**	0.00	1.23 ± 0.58	10.898 *	<0.001
**AFID (mg/g)**	0.00	1.53 ± 0.83	8.843 *	<0.001

Legend: m ± s—mean ± standard deviation; t—test statistic in t-Student test for independent samples; t*—test statistics in t-Student test for single sample; *p*—statistical significance; CC—costal cartilage; AFID—annulus fibrosus of the intervertebral disc. The test statistics derived from this analysis are indicated by an asterisk.

**Table 3:** Changes in blood ethyl alcohol concentration over time in individuals classified as sober.

Time [Days]	Blood [mg/mL]			Limit 0.5‰		Limit 0.0‰	
	Me (Q_1_–Q_3_)	min	max	z	*p*	z	*p*
0	0.000(0.000–0.000)	0.000	0.450	5.31	<0.001	0.89	0.167
1	0.000(0.000–0.000)	0.000	0.280	1.98	<0.05	0.0	0.050
2	0.080(0.000–0.100)	0.000	0.220	1.90	<0.05	1.34	0.091
3	0.000(0.000–0.000)	0.000	0.040	1.97	<0.05	0.00	0.050
4	-	0.000	0.000	-	-	-	-
7	-	0.000	0.000	-	-	-	-

Legend: Me (Q_1_–Q_3_)—median (first quartile–third quartile); z–Wilcoxon (signed-rank) test statistic; *p*—statistical significance.

**Table 4:** Changes in ethyl alcohol concentration in urine, CC, and AFID over time in individuals classified as sober.

Time [Days]	Urine [mg/mL]			CC [mg/g]			AFID [mg/g]		
	Me (Q_1_–Q_3_)	min	max	Me (Q_1_–Q_3_)	min	max	Me (Q_1_–Q_3_)	min	max
0	0.000 (0.000–0.000)	0.000	0.340	0.000 (0.000–0.000)	0.000	0.230	-	0.000	0.000
1	0.000 (0.000–0.130)	0.000	0.520	0.000 (0.000–0.000)	0.000	0.150	-	0.000	0.000
2	0.000 (0.000–0.128)	0.000	0.510	0.000 (0.000–0.000)	0.000	0.110	-	0.000	0.000
3	0.000 (0.000–0.270)	0.000	0.540	-	0.000	0.000	-	0.000	0.000
4	0.000 (0.000–0.225)	0.000	0.450	-	0.000	0.000	-	0.000	0.000
7	0.000 (0.000–0.125)	0.000	0.250	-	0.000	0.000	-	0.000	0.000

Legend: Me (Q_1_–Q_3_)—median (first quartile–third quartile); CC—costal cartilage; AFID—annulus fibrosus of the intervertebral disc.

**Table 5:** Statistically significant differences in blood ethyl alcohol concentration in intoxicated individuals over time.

Blood		m ± s	min	max	Limit 0.5‰		Limit 0.0‰	
Status	Storage Time After Sample Collection [Days]				t	*p*	t	*p*
intoxicated	0	2.792 ± 1.191	0.980	5.800	10.00	<0.001	12.18	<0.001
	1	2.320 ± 1.012	0.710	4.800	9.34	<0.001	11.91	<0.001
	2	1.582 ±0.832	0.000	2.830	6.76	<0.001	9.88	<0.001
	3	0.890 ± 0.743	0.000	2.500	2.73	<0.05	6.22	<0.001
	4	0.450 ±0.479	0.000	1.740	0.55	0.589	4.88	<0.001
	7	0.114 ± 0.340	0.000	1.610	5.90	<0.001	1.75	0.093

Legend: m ± s—mean ± standard deviation; t—t-Student test statistic for one sample; *p*—statistical significance.

**Table 6:** Detailed comparisons of ethyl alcohol concentrations across consecutive study days in intoxicated individuals.

	Day	1	2	3	4	7
Blood	0	<0.001	<0.001	<0.001	<0.001	<0.001
	1		<0.001	<0.001	<0.001	<0.001
	2			<0.001	<0.001	<0.001
	3				<0.001	<0.001
	4					<0.001
Urine	0	<0.001	<0.001	<0.001	<0.001	<0.001
	1		<0.001	<0.001	<0.001	<0.001
	2			<0.001	<0.001	<0.001
	3				<0.001	<0.001
	4					<0.001
CC	0	<0.001	<0.001	<0.001	-	-
	1		<0.001	<0.001	-	-
	2			0.073	-	-
AFID	0	<0.001	<0.001	<0.001	-	-
	1		<0.001	<0.001	-	-
	2			0.702	-	-

Legend: CC—Costal Cartilage; AFID—Annulus Fibrosus of the Intervertebral Disc.

**Table 7:** Changes in ethyl alcohol concentration by time point and matrix type in intoxicated individuals over time.

Matrix/Mould	Time [Days]	m ± s	min	max	Limit 0.0‰	
					t	*p*
Urine [mg/mL]	0	3.246 ± 1.695	0.310	6.300	8.74	<0.001
	1	2.442 ± 1.442	0.710	5.300	7.76	<0.001
	2	1.988 ± 1.326	0.000	4.440	6.87	<0.001
	3	1.569 ± 1.207	0.000	4.160	5.95	<0.001
	4	1.231 ± 1.003	0.000	3.560	5.48	<0.001
	7	0.573 ± 0.732	0.000	2.20	3.32	<0.01
CC [mg/g]	0	1.233 ± 0.577	0.220	2.300	10.90	<0.001
	1	0.737± 0.448	0.000	2.000	8.38	<0.001
	2	0.286 ± 0.335	0.000	1.440	4.35	<0.001
	3	0.075 ± 0.231	0.000	1.130	1.66	0.109
	4	0.000 ± 0.000	0.000	0.000	-	-
	7	0.000 ± 0.000	0.000	0.000	-	-
AFID [mg/g]	0	1.538 ± 0.834	0.220	2.300	8.84	<0.001
	1	1.031± 0.778	0.000	2.000	6.36	<0.001
	2	0.196 ± 0.421	0.000	1.440	2.24	<0.05
	3	0.029 ± 0.136	0.000	1.130	1.00	0.328
	4	0.000 ± 0.000	0.000	0.000	-	-
	7	0.000 ± 0.000	0.000	0.000	-	-

Legend: m ± s—mean ± standard deviation; min—minimum value; max—maximum value; t—test statistic from the one-sample *t*-test; *p*—statistical significance.

## Data Availability

The raw data supporting the conclusions of this article will be made available by the authors on request.

## Supplementary Materials

The following supporting information can be downloaded at https://www.mdpi.com/article/10.3390/toxics14080720/s1, Tables S1: Sex-based comparison of the analysed variables at (t0); Table S2: Univariable regression analysis of ethyl alcohol concentrations in blood, urine, CC, and AFID among intoxicated individuals; Figure S1. Ethyl alcohol concentrations in (a) blood and urine, and (b) in costal cartilage (CC) and Annulus Fibrosus of the Intervertebral Disc (AFID), including the experimental data. The same scale was used for both graphs.

## Abbreviations

The following abbreviations are used in this manuscript in alphabetical order:

AFID	annulus fibrosus of the intervertebral disc
As	skewness coefficient
β	regression coefficient
χ^2^	chi-squared test
CC	costal cartilage
EtOH	ethyl alcohol
GC-FID	gas chromatography with flame ionization detection
GenAI	generative artificial intelligence
GTFCh	German Society of Toxicological and Forensic Chemistry
m	mean value
m ± s	mean ± standard deviation
max	maximum value
Me	median
Me(Q_1_–Q_3_)	median (first quartile–third quartile)
min	minimum value
N	number of cases
*p*	*p*-value, probability/statistical significance
PMI	post-mortem interval
Q_1_	lower quartile, first quartile
Q_3_	upper quartile, third quartile
r	Pearson’s correlation coefficient
r_S_	Spearman’s rank correlation coefficient
s	standard deviation
SE(β)	standard error of the regression coefficient
T	test statistics
V_s_	coefficients of variation
z	test statistics
