# Supplementary material for: Can Post-Mortem Cartilage Reliably Preserve Ethanol? A Time-Course Comparison with Blood and Urine
Source: Toxics. 2026 Aug 14;14(8):720. doi: 10.3390/toxics14080720 (PMC13517706; doi:10.3390/toxics14080720)
Supplement: Supplementary file 1 [file toxics-14-00720-s001.zip › toxics-4473180-supplementary.pdf]

Supplementary Table S1. Sex-based comparison of the analysed variables at (t0)

Variable	Sex		t/z	p
	Female N=12	Male N=45		
Age (years)	35.8 ±15.6	43.5±16.1	1.451^t	0.153
PMI (days)	2.5 (2.0-3.5)	4.0 (3.0-5.0)	1.935^z	0.053
Ethyl alcohol concentration in:				
Blood (mg/ml)	0.00 (0.00-2.69)	0.45 (0.00-2.52)	0.500^z	0.617
Urine (mg/ml)	0.00 (0.00-3.17)	0.56 (0.00-3.04)	0.590^z	0.555
CC (mg/g)	0.00 (0.00-1.31)	0.22 (0.00-1.13)	0.241^z	0.810
AFID (mg/g)	0.00 (0.00-1.53)	0.00 (0.00-1.36)	0.059^z	0.953

Legend: PMI – Post Mortem Interval; t – test statistics in t-Student test for independent sample, z – Mann–Whitney U test statistic; p – statistical significance, CC – Costal Cartilage, AFID – Annulus Fibrosus of the Intervertebral Disc, M ±SE - mean = standard deviation, Me(Q1-Q3) - median (lower - upper quartile)

Supplementary Table S2. Univariable regression analysis of ethyl alcohol concentrations in blood, urine, CC, and AFID among intoxicated individuals.

Blood	β	SE(β)	r	p
**Urine**	1.063	0.215	0.750	<0.001
**CC**	0.418	0.050	0.861	<0.001
**AFID**	0.570	0.074	0.859	<0.001

Legend: β – regression coefficient; SE(β) – standard error of the regression coefficient; r – Pearson's correlation coefficient; p – statistical significance; CC – Costal Cartilage, AFID – Annulus Fibrosus of the Intervertebral Disc.

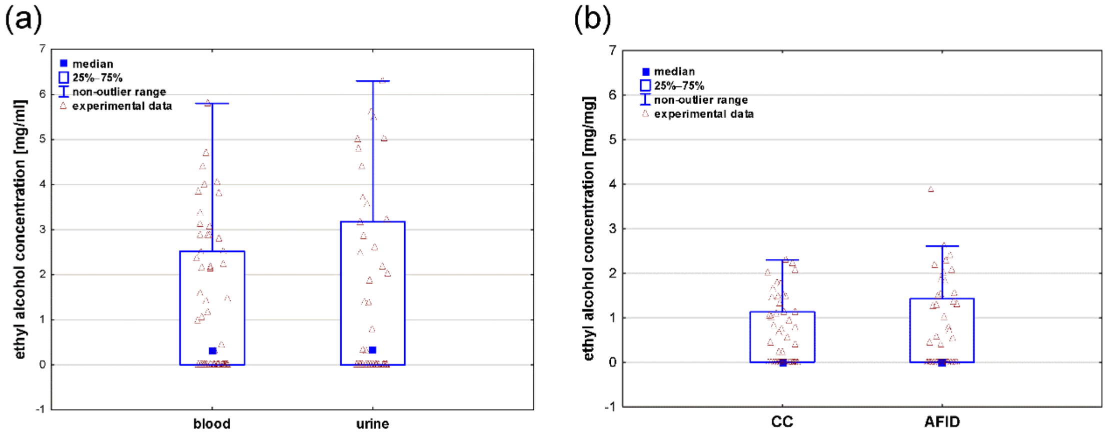

Supplementary Figure S1. Ethyl alcohol concentrations in (a) blood and urine, and (b) in costal cartilage (CC) and Annulus Fibrosus of the Intervertebral Disc (AFID), including the experimental data. The same scale was used for both graphs.
